# Valuing health states for infants and toddlers: challenges and methodological considerations in EQ-TIPS preference elicitation

**DOI:** 10.1007/s10198-025-01789-0

**Published:** 2025-07-18

**Authors:** Nancy Devlin, Bram Roudijk, Michael Herdman, Elly Stolk, Richard Norman, Simone Kreimeier, Janine Verstraete

**Affiliations:** 1 Melbourne School of Population and Global Health, University of Melbourne, 207 Bouverie Street, Parkville, Melbourne, VIC 3010 Australia; 2 EuroQol Research Foundation, Rotterdam, The Netherlands; 3 Saw Swee Hock School of Public Health, National University of Singapore, Singapore, Singapore; 4 Curtin University, Bentley, Australia; 5 Bielefeld University, Bielefeld, Germany; 6 University of Cape Town, Cape Town, South Africa

**Keywords:** Pediatrics, HRQoL, Child QALYs, Utilities, Values, Young children, I00, I31

## Abstract

Many health care interventions are aimed at very young children, including public health measures such as vaccines, and new, high-cost medicines for rare diseases. This has led to increasing interest in evidence on the effectiveness and cost effectiveness of treatments in this age group. EQ-TIPS has been developed as a concise, generic health outcomes measure in 0–3-year-olds. Preference weights for EQ-TIPS are required to facilitate its use in economic evaluation. The aim of this paper is to consider the issues likely to be encountered in attempts to elicit stated preferences for EQ-TIPS. We begin by identifying the challenges that would arise if the existing EQ-5D-Y-3L (‘Y-3L’) valuation protocol were used as a starting point to value EQ-TIPS states. We highlight the challenges specific to valuing EQ-TIPS *over and above* the challenges already noted in valuing the Y-3L. We then discuss broader issues that may arise in valuing EQ-TIPS: (a) the potential influence on values of respondents taking into account the future consequences of developmental delay arising from poor health in infants, and (b) spill-over effects of poor health in infants on parents/caregivers. Methods used in valuing other instruments in this age group are reviewed. We conclude that eliciting stated preferences for EQ-TIPS would require adapting existing valuation methods. Parent-based valuation may be a viable approach, although methodological complexities remain. Alternative means of preference weighting EQ-TIPS, including mapping to the EQ-5D-Y, may offer a way forward.

## Introduction

The topic of valuing health states generated by generic measures of child health-related quality of life (HRQoL), such as EQ-5D-Y-3L and CHU9D, has been the subject of considerable debate and research in recent years [1, 2]. While methodological challenges remain [3–5], considerable progress has been made in better understanding and addressing the issues, building an evidence base on the characteristics of values for child HRQoL, and in providing value sets to support the use of pediatric instruments in economic evaluation [6, 7].

In contrast, the question of how to value HRQoL in infants and toddlers (for the purposes of this paper, defined as 0–3 year olds) – children much younger than the age-groups covered by most available preference-weighted generic measures of pediatric HRQoL [8] – remains relatively uncharted territory. This is an important gap in methods and evidence because many health care interventions are aimed at very young children. Over a third of pediatric cost effectiveness analyses focus on interventions for infants and toddlers [9]. These factors have led to increasing interest in evidence on the effectiveness and cost effectiveness of treatments in this age group. However, evidence on HRQoL submitted to Health Technology Assessment (HTA) bodies for technologies aimed at very young children has been shown to be sub-optimal in some cases. A 2021 review of NICE decisions concerning preschool children showed that most used generic HRQoL measures and values designed for adults [10]. The authors noted that appraisal committees frequently expressed concern about the limitations in HRQoL data and utilities in this age range and recommended that research on valuation of measures for younger children should be a priority [10]. The use of utilities for adult HRQoL has also been noted in a review of evidence submitted to PBAC regarding pediatric interventions. [11]

Instruments which are available for use in younger children, and for which preference weights are available, include the Health-Related Quality of Life Utility Measure for Pre-School Children (HuPS) [12, 13]. HuPS was designed for use as a stand-alone measure of HRQoL for children aged 2 to 4 years of age, while being capable of generating utilities that are ‘commensurate and continuous with’ the utilities for children and adults produced by the Health Utility Index (HUI3). The HuPS classification system was based on the HUI3, and Health Status Classification System for Preschool children (HSCS-PS), designed for 2.5 to 5 year olds [12]. Methodologists, paediatricians and parents reduced the 12 items and corresponding levels of the HSCS-PS to 8 items and the corresponding levels of report of the HUI3. This resulted in the new HuPS measurement system, comprising 8 attributes, with 3 to 6 levels of report, including: vision, hearing, speech, ambulation, dexterity, emotion, cognition, and pain and discomfort. Parents of children aged 2 to 6 years from the general population, pediatricians and researchers paired the HuPS level descriptors to a HUI3 level descriptor which has a pre-existing utility coefficient [13]. These methods enabled HuPs health states to be preference-weighted using existing HUI3 utilities, rather than basing utilities on preferences specific to the age range covered by HuPs.

Other efforts to provide instruments which can be used in this age group have involved the adaptation of instruments initially developed and validated for older children – CHU9D and EQ-5D-Y-3L (‘Y-3L’) – for use in younger children; in both cases, for 2–4 year olds [14–16]. Qualitative research has been undertaken to explore valuation issues relating to the adapted version of Y-3L [17], including the feasibility of using latent scale discrete choice experiments (DCE) tasks.

Two instruments developed specifically for younger children have been published recently to support assessments and potential for valuation of HRQoL in this age group: the Infant health-related Quality of life Instrument (IQI) [18–20], and the Toddlers and Infants (TANDI) measure [21, 22], subsequently renamed EQ-TIPS (EuroQol—Toddler and Infant Populations).

The IQI was developed in a multi-country study (China, Hong-Kong, UK, USA, New Zealand, Singapore) and is recommended for use in infants aged 1–12 months. It includes 7 items: sleeping, feeding, breathing, stooling/poo, mood, skin, and interaction. Each item consists of 4 levels, most of which are ranked by severity. The IQI is accompanied by a preference-based scoring system. These were initially generated using latent scale discrete choice experiments [19] and, later, via a two-step procedure involving a DCE which included comparisons against ‘dead’, yielding values anchored at 0 and 1 as required for quality-adjusted life year (QALY) estimation [20].

EQ-TIPS [21, 22] is available in both three and five level versions. Version 3 comprises seven dimensions (movement, eating or drinking, sleep, pain, managing emotions, interacting with others, play) Its three-level variant uses no, some or a lot of problems (see Appendix 1); its five-level variant uses no, a little bit, some, a lot and extreme problems. EQ-TIPS is a member of the EuroQol Group’s broader family of instruments, which includes the Y-3L [23] and EQ-5D-Y-5L (‘Y-5L’) [24] for children, and the EQ-5D-3L [25] and EQ-5D-5L [26] for adults. EQ-TIPS is currently an experimental instrument, as evidence builds to support its use and to establish its psychometric properties [27], but there has nevertheless been considerable interest from researchers to use it in diverse language, cultural, and clinical contexts. Applications have to date been approved for its use in disease registries, studies into the impact of Covid-19, genetic screening, and the measurement of HRQoL in specific disease groups.

Value sets are currently not available for EQ-TIPS, though the intention is to eventually provide a means of preference weighting the data generated from it to facilitate QALY estimation. Nevertheless, the fact that the instrument is designed for use in very young children and over a relatively short age range (0–3 years) poses challenges that may not be present when valuing health states in older children or adults.

The aim of this paper is to explore those challenges and consider some of the ways in which they might be addressed. As a starting point, we begin by considering whether the existing protocol used to value the Y-3L [28] could be applied, as it stands, to the valuation of EQ-TIPS, and what drawbacks there might be to that approach. We use this approach as a means of identifying any potential challenges specific to valuing EQ-TIPS *over and above* those that already exist when valuing health states for older children. We then consider means of addressing those challenges, including by examining valuation methods used by the developers of other instruments designed for use in this age group. We then discuss broader issues that may arise in valuing EQ-TIPS. We conclude by considering a) whether it is necessary and feasible to elicit age-specific values for EQ-TIPS health states, b) the range of methods potentially available to do so, and their pros and cons, and c) whether there are other ways of valuing EQ-TIPS.

While this paper is motivated by our consideration of issues related to the valuation of EQ-TIPS, these same issues and considerations are arguably relevant to any attempt to value HRQoL in these youngest age groups.

## Issues with use of existing valuation methods to value HRQoL in infants and toddlers

Valuation of pediatric HRQoL health states raises complex methodological and normative issues [2, 29] i.e. methods choices that involve value judgements. These include whose stated preferences are sought (e.g. adults or children), and, where adults’ preferences are sought, what perspective they are instructed to adopt, and what age of child is considered. Research addressing those questions have included valuation of vignettes and generic pediatric HRQoL instruments [30], such as the Y-3L [23] and CHU9D [31], which were developed and validated for older children e.g., in the case of the Y-3L, for children aged 8 or over (although a proxy version is available for use in children > 4 years of age).

Most of the valuation issues that have been identified in relation to these pediatric instruments [1, 2] *also* apply to valuation of HRQoL in younger children. However, the valuation of health states relating to infants and toddlers presents additional challenges, some of which are novel, and some of which are more extreme versions of similar issues encountered in valuing HRQoL in older children.

To illustrate, consider the methods currently included in the international valuation protocol for Y-3L [28]. Table 1 summarises the key elements of that protocol and highlights the issues and adaptations to those methods that may be required if these methods were considered as a starting point for use in valuing HRQoL in infants and toddlers. We discuss each, including a summary of how each issue has been handled in valuation of IQI and HuPS.

**Table 1:** Key elements of the protocol used in valuing EQ-5D-Y-3L, and summary of issues arising with application of those methods to value EQ-TIPS

Key protocol feature	EQ-5D-Y-3L	Application to valuation of EQ-TIPS?
*(a)Whose values are relevant?*	**Adult general population**	**Adult general population** • It may be more difficult for members of the adult general population to conceptualise and evaluate health states in infants and toddlers than in older children. Adults may be able to recall their own experiences as a child, but typically not as an infant. Further, while EQ-5D-Y-3L dimensions closely overlap with descriptions of adult EQ-5D states, that is not so much the case with EQ-TIPS • Parental/caregiver preferences may be more relevant for the valuation of health states in very young children e.g. because of their current experience with children in the EQ-TIPs age range • If parental/caregiver preferences are considered relevant, should they be the *only* group sampled, or a quota within a general public sample?
	• Some studies have additionally sought adolescents’ preferences for EQ-5D-Y-3L	• In contrast to EQ-5D-Y-3L, there is no possibility of obtaining stated preferences from children in the age range covered by EQ-TIPS. Proxy valuation is unavoidable
*(b) What perspective?*	**Adults, considering their views regarding health states in a hypothetical child** • Adolescents’ preferences may be obtained, in which case the perspective used is typically their ‘own health’	**‘Hypothetical child’** • Asking adults to imagine health states as experienced by a hypothetical infant is arguably a more difficult task (compared to imagining EQ-5D-Y-3L states being experienced by an older child) since we cannot recall our experience as an infant, or readily imagine how an infant experiences such problems • If the preferences of parents of infants/toddlers are considered relevant, the most suitable perspective may be ‘own child’
**Note:** Asking adults to value EQ-5D-Y states from their *own* perspective (blinded to the states being child-specific) is possible in principle and, while not part of the Y-3L valuation protocol, has been used eg. valuation of CHU9D [30]. In contrast, asking adults to value EQ-TIPS states from a ‘self’ perspective is not possible because the nature of the items means it is impossible to ‘blind’ them to the states relating to infants		
*(c) Age of child?*	**10 years of age [Age covered by EQ-5D-Y-3L: 8–15 years]**	**Age covered by EQ-TIPS: 0–3 years** • Following the EQ-5D-Y-3L valuation protocol would suggest selecting a single age within the age range covered by EQ-TIPS e.g. 1 year old. Although the age range covered by EQ-TIPS is relatively narrow, development during infancy is rapid. Research can test the impact on values of choice of age • If parents/caregivers value EQ-TIPS states considering their own child, the question of age can be resolved by seeking a sample of parents/caregivers with children across the 0–3 year age range
*(d) Methods?*	**DCE (*n* = 1000) + cTTO (*n* = 200) to anchor**	**DCE + cTTO?** • DCEs which do not include ‘dead’ or duration are non-contentious and can provide useful information on the relative importance of dimensions. However latent scale DCE does not yield values anchored on a 0–1 scale, so an additional step would be required to enable anchoring • Anchoring latent scale preferences for EQ-TIPS using cTTO, as in the EQ-5D-Y-3L protocol, raises a number of additional challenges—see (e)
*(e) Duration of states?*	**DCE: Duration not specified** **cTTO:** 10 years (chosen for consistency with methods used to value adult HRQoL)	**DCE: Duration not specified** **cTTO:** A 10 year duration is precluded as it spans life years well beyond the age group relevant to EQ-TIPS • EQ-TIPS items are much less relevant to children > 3 years, so asking respondents to imagine those states to have durations that go beyond 3 years of age makes no sense • A 10 year duration of an EQ-TIPS state would mean the scenario being considered is dominated by years outside the 0–3 age range **Solutions could include:** • Opting for much shorter (e.g. 1-year) duration. However, this may cause issues with task acceptability and non-trading • Retain the 10 year duration, but create a ‘composite’ scenario e.g. an EQ-TIPS state for x years, followed by an ‘equivalent’ EQ-5D-Y-3L state for (10-x) years. The valuation of such a composite state would raise new challenges in designing the task and interpreting results • Anchoring stated preferences for EQ-TIPS using anchors obtained for other EQ instruments (eg. EQ-5D-Y-3L) e.g. via mapping

### Whose stated preferences should be sought to value EQ-TIPS

The protocol for valuing the Y-3L recommends preferences be sought from adult members of the general public. Eliciting *adults’* preferences for child health states was considered to be broadly consistent with the way adult HRQoL is valued [28] and broadly in keeping with requirements of a number of HTA bodies, such as NICE [32]. Inherent to this approach is a value judgement that it is adult members of the general public whose preferences are relevant, as taxpayers and potential patients [33]. Some Y-3L valuation studies have additionally sought stated preference data from older children, for comparative purposes, arguing there is normative support for obtaining preferences from those whose health is being described (e.g. ‘nothing about me without me’) [1]. This appears to also command support from decision makers and other stakeholders [34–36]. Notably, in the case of EQ-TIPS, it is not possible to obtain preference information from *any* child within the age range for which the instrument was designed (0–3 years old), so this option is ruled out. Given that valuation from an ‘own health’ perspective is precluded, who is best placed to value EQ-TIPS states, and why? In part, the answer to this question depends on what exactly we instruct the ‘valuer’ to do: are we asking them for their *own* preferences regarding the health of an infant? (the approach currently used in Y-3L valuation; which Lipman et al. (2022) [37] calls the ‘Proxy 1 perspective’), or are we asking them to tell us what they believe the preferences of an infant/toddler to be (‘Proxy 2 perspective’)?

Both Proxy 1 and Proxy 2 valuation of EQ-TIPS rely on a proxy respondent being able to conceptualise and imagine the experience of an infant/toddler living in the stylized health states described by EQ-TIPS. This may be more feasible for those who are currently parents or caregivers of infants and toddlers, than for other adults.^1^ Parents of infants and toddlers have intense current experiences of living with and caring for children in that age range. That experience may be important, because whereas most adults have *some* recollection of what it was like to be a child themselves, and can in principle draw on those experiences to value child HRQoL (e.g. as described by Y-3L), arguably *no* adult can recall their ‘lived experience’ of being an infant. Most parents (e.g. of older children and children who are now adults) will have some recollection of the experience of caring for an infant, but that recollection may differ depending on how recent that experience was. The accuracy of recalled experience of parenting infants and toddlers may decline over time, though that experience may be ‘topped up’ again by new experiences of caregiving in that age range e.g., as grandparents. The argument that parents of 0–3 year olds are better placed to value EQ-TIPS because of their current ‘lived experience’ has some similarity to the arguments in favour of patients’ (rather than general public) valuation of adult health states. An important distinction is that the call for patient preferences is often rejected on the grounds that the health care system is concerned with all patients and potential patient preferences, not just those whose treatment is currently under consideration, and that the general public represents taxpayers who fund health care. In contrast, inviting parents of young children to value EQ-TIPS states is not suggested to be restricted to parents of young children who are currently ill; and parents in the general public are also taxpayers.

The Proxy 2 perspective on valuation of EQ-TIPS is problematic for other reasons. In effect, Proxy 2 requires that the respondent is able to accurately reflect the preferences of an infant.^2^ It is not clear to what extent a 0–3 year old is ‘aware’ of their health state, or the possibility of other states, or to have preferences regarding them.^3^ If infants and toddlers do not have preferences, then it seems contradictory to ask a parent or other adult to respond to stated preferences tasks in a way consistent with them. By this logic, a Proxy 2-type valuation of EQ-TIPS is probably ruled out.^4^

This leaves us with Proxy 1 valuation, either by members of the adult general public or parents/caregivers. As argued above, parents/caregivers are better able to conceptualise and imagine the states to be evaluated. The experiences of infants and toddlers, and states described by EQ-TIPS, may be too far removed from the point of reference of most adult members of the general public. Further, parents may have more of a ‘moral claim’ to valuing EQ-TIPS states, as they make *all* decisions for infants; valuation of EQ-TIPS might be regarded as an extension of that role. Van Heusden et al. (2024) report broad support for asking parents to value health states in 2–4 year olds [17]. Asking parents/caregivers of infants/toddlers is, however, a departure from the usual approach to health state valuation, whereby values represent the average preferences of a society, not the preferences of sub-groups within it.^5^ There is limited evidence on how parents and non-parents’ preferences differ in relation to the health states of 0–3 year olds. However, Xiong et al. [38] report that preferences for CHU9D states in 2-4 year olds differed between parents and non-parents. 

Contrasting approaches to this question of whose preferences are relevant are taken in the valuation of IQI and HuPs. In preference-weighting IQI, initial DCE work included primary caregivers of children 0–3 years [19] and subsequent DCE and Best Worst Scaling (BWS) included primary caregivers of children aged 0–3 years and members of the general population [20]. The authors assumed that caregivers of children aged 1–3 years could recollect their child’s past experiences. Adults of the general population were subsequently included as it was postulated that they may have different views [20]. HuPS used parents of children aged 2 to 6 years from the general population to represent the community perspective in undertaking the item matching tasks that were used to create the link between HuPS and HUI3 [13]. The rationale for the extended age range (i.e. older than the age of children whose health is measured by HuPS) was not provided. Note however that once the link between HuPS and HUI3 descriptive systems is established, it is utilities for the latter that are used to preference weight both. That is, preferences of the adult general public, not specific to the age of children whose states are measured via HuPS.

### From what perspective are respondents asked to value health states?

The question of whose preferences are relevant is closely related to the question of what perspective respondents are asked to adopt in valuing health states. If parents/caregivers of children in the age group in question are asked about their health state preferences considering their own child, the question of perspective is easily solved (‘When thinking about your child, ….’). However, if respondents in the valuation task are adult members of the general public, different options are possible. In the case of the Y-3L, adult respondents are asked to think of a 10 year old child when valuing health states, though in principle they could instead be asked to value the states from an ‘own health’ perspective i.e. blinded to the states under consideration being specific to children, and imagining themseves living in the states described. This was the approach taken to valuing CHU9D in the UK [31]. In the case of EQ-TIPS, such an approach is ruled out: the health state descriptions are clearly specific to very young children. The remaining options are therefore to ask respondents to imagine a hypothetical infant/toddler, or (in the case of parents) their own child as an infant/toddler. In the case of the IQI, adults from the general public were asked to consider ‘an infant’s first year of life’ [20].

Of course, asking people to value health states for others (a ‘third person’ approach) is very different to the approach usually taken for health states generated by instruments for adults, such as EQ-5D-5L [39], where people are asked to value health states for themselves (a ‘first person’ approach). This can potentially lead to greater reluctance to trade, because respondents are trading the life years of another person [40]. This effect can be exacerbated further when valuing the health of children, e.g. in the case of time trade-off (TTO), by a greater reluctance to trade-off life years than is the case when adult health states are under consideration [5]. These issues are potentially even more relevant to EQ-TIPS than to Y-3L, given the even shorter life of the children whose health scenarios are being considered.

### What age of child to specify?

Where adult respondents are asked to value states of a hypothetical child (rather than their own children), it can be helpful to specify the age of child to be considered. In the case of the Y-3L, this was set at 10 years of age. The values obtained are then assumed to be applicable to health states of children both older and younger than 10 years, within the age range covered by the instrument. The evidence on whether specified age exerts an effect on values is mixed – there is some evidence that age might matter in obtaining TTO values [41] and other evidence suggesting that it has no effect on latent scale DCE preferences [42] although in both cases the ages considered were children older than the age range covered by EQ-TIPS. In the case of EQ-TIPS, the age range covered by the instrument is relatively narrow at 0–3 years. On the other hand, development occurring during these years is considerable—and the relative importance of dimensions may differ within this age range e.g. play, communication and social interactions may be considered much more important in a 3 year old than in a newborn infant. Age could be fixed at a single age e.g. 1 year old, or could be varied e.g. newborn, 1, 2 and 3 years old, to test whether specified age is important. Van Heusden et al. (2024) report that participants valuing the Y-3L for 2–4 year olds stated that there would be no difference in their responses if considering a 2 vs. a 4-year-old [17]. A recent study that randomized respondents to consider hypothetical health states for children of ages 2, 3, 4, or 5 found no systematic significant differences in health state values across these ages [43]. In the case of IQI, the age range of the instrument and thus the age to specify in stated preferences tasks was perhaps easier and is referred to as ‘the first year of life’ [20].

If the characteristics of values for EQ-TIPS do differ by age, this leaves a conundrum: do we overlook it by choosing a single age, or (more controversially, and more costly) produce different values for each age 0–3 years? Overlooking evidence of preference differences by age is a pragmatic solution, and is the standard approach taken for existing pediatric and adult instruments; changing that approach in this population would require a strong rationale.

As noted earlier, if we ask parents/caregivers of infants/toddlers for their preferences, the age of child considered in each case would be that of their own child, rather than being pre-specified by researchers. In this case, it would be important to ensure the sample evenly covers parents/caregivers of children in the 0–3 year age range.

### Which stated preference methods to use and (e) what duration of states?

A bigger issue, in terms of needing to deviate from the valuation practices used in valuing the Y-3L, arises in relation to duration of the EQ-TIPS states to be valued. This, in turn, is linked to the question of what methods to use to elicit preferences.

By convention, the duration of states in valuation tasks is 10 years. Although arbitrary, the use of a fixed 10-year duration in TTO tasks is an accepted part of the standard methods used in valuing both the adult EQ-5D-5L and the Y-3L. The use of a 10-year duration when applied to the valuation of Y-3L states experienced by a 10-year old have been noted elsewhere to involve imagining health states beyond childhood [1, 6].

The 10-year duration becomes significantly more difficult if applied to valuing EQ-TIPS. This is both because (i) the narrower age range to which EQ-TIPS pertains means that a 10-year duration of an EQ-TIPS state would mean the scenario being considered is dominated by years outside the 0–3 age range; and (ii) the EQ-TIPS health state descriptions are not appropriate or relevant for older children. The IQI avoided this issue by not stating a duration in the DCE tasks; anchoring of values used best worst scaling (BWS) and, rather than states being followed by death, the tasks specified ‘health is uncertain afterwards’ [19, 20]. In the case of HuPS, the issue is avoided by not eliciting age-specific values at all but mapping to HUI3 and using its preference weights [13].

The question of whose stated preferences are sought, from what perspective and for what age of child are unavoidable regardless of what method is used to elicit stated preferences for EQ-TIPS. Use of latent scale DCE would avoid the complexities around duration noted above, as duration is typically not specified in such tasks. Age of child would, however, still need to be specified (other than in the case of parents considering their own infant/toddler). DCE provides a ready means of establishing the relative importance of the dimensions. However, latent scale DCE does not produce values anchored at 0 and 1 as required for QALY estimation. The use of TTO invokes the more complex considerations noted in Table 1.

The application of TTO to EQ-TIPS may encounter issues of acceptability to respondents, especially if using shorter time frames such as 1 year, after which the infant/toddler will die. Issues with unwillingness to trade and non-trading behaviour, already noted in Y-3L valuation, are likely to be exacerbated. This was the experience of Matza et al. (2025) who found a 1-year time horizon problematic when used in pilot valuation of toddler health states relating to pneumococcal disease, resulting in minimal differentiation among health states and reluctance to sacrifice any time from the short lifespan [43]. One option would be to retain the 10 year duration, but create a scenario which is an amalgam of EQ-TIPS and Y-3L states e.g. an EQ-TIPS state for *x* years, followed by an ‘equivalent’ Y-3L state for (10-*x*) years. Designing these composite states relies on being able to establish descriptively equivalent states, and on the assumption that the shift between health state descriptions does not lead to violations of additive independence (i.e. if the values for EQ-TIPS states are influenced by the selection of Y-3L states to follow it). The requirement to explain the different states in a composite scenario may also be challenging for respondents to comprehend. The valuation of EQ-TIPS via inclusion in an amalgam scenario therefore presents numerous challenges. These may be possible to overcome but represent a departure from ‘standard’ methods and may create a challenge in interpreting and comparing values eg. with values for Y-3L or EQ-5D-5L.

While we have focused our consideration of the challenges involved in valuing EQ-TIPS to the methods currently employed in the Y-3L valuation protocol, it should be noted that other stated preference methods are available e.g. Standard Gamble; Best Worst Scaling; DCEs incorporating duration attributes. The issues we have identified with respect to whose preferences should be elicited; what perspective shoud be taken by respondents in valuing the health states in this age range; and how to handle duration of states to be evaluated, would also be encountered in the use of these methods to produce values anchored at 0 and 1 for the purposes of QALY estimation.

## Further challenges in valuation of toddler and infant health states

Table 1 focuses on specific issues that would be associated with the use of the existing protocol for valuing Y-3L to value EQ-TIPS. Clearly, the elicitation of values for EQ-TIPS states could not proceed using the protocol used to value Y-3L without considerable changes. Moreover, there are further issues that may be relevant to consider in valuation of EQ-TIPS.

Firstly, valuation of HRQoL states in infants and toddlers may be extremely difficult to disentangle from considerations about the future health and non-health consequences of poor HRQoL in younger years, around which consquences there may be considerable uncertainty. Valuation of HRQoL in very young children may be dominated not by imagining what it would be like for an infant or toddler to experience that state *now*, but by broader considerations regarding the consequences of poor HRQoL now in terms of delayed or impaired child development and the consequences thereof in future. These include future health, social, educational, employment and socioeconomic considerations. For example, neonatal sepsis is associated with longer term health impacts including cerebral palsy, respiratory problems, cognitive delay, psychomotor delay and auditory impairment [44]. These long term effects are important to incorporate into economic models for interventions to prevent or treat neonatal sepsis. However, the internalization of such concerns into the valuation of the child health state may lead to double-counting of these longer-term consequences. Similarly complex and wide-ranging long term effects may result from other neonatal disorders. If these future prospects, and/or the attitudes to risk surrounding them, dominate responses to the valuation task, the approach to valuing HRQoL in infant and toddlers, and interpretation of values for estimating QALYs, may require a fundamental re-think.

Secondly, given that children in the EQ-TIPS age range are altricial (i.e. entirely or highly dependent), the spillover effects of poor health in this age group are likely to be linked to the health and wellbeing of parents and caregivers [45–48] and, potentially, siblings. Therefore, we need to address either how that is accounted for or how to disentangle it from values for EQ-TIPS states. Such effects can, in principle, be accounted for separately in QALY estimates eg. via the use of an instruments to measure carer impacts such as the CarerQol [49]. A recent Taskforce recommends explicit inclusion of such spillover effects into economic evaluation of pediatric interventions [50]. A key consideration in valuing of EQ-TIPS states by either parents or other adults would therefore be ensuring that such considerations do not also influence their valuation of the states. However, identifying a framing that isolates the health of the child from that of the parent is carer is difficult given how closely linked the two are in the early years of life.

The issues above have also been noted as a factor with respect to valuing older children’s health and questions raised about how best to address broader factors, such as lifecourse distributional effects, in cost effectiveness analysis of childhood interventions [51]. At present, there is very little evidence regarding the factors that adult respondents take into account when considering health states in very young children. We hypothesise that the broader factors outlined above may play an important role in valuing EQ-TIPS, and to a greater extent than in valuation of Y-3L. This could be tested empirically, both through qualitative think-aloud studies, and through quantitative methods observing the effect of more explicitly controlling for these spillovers in a valuation context.

Research to identify whether values elicited for HRQoL states in infants and toddlers are affected by respondents taking into account such spill over effects is needed to ensure the comparability of values and QALY estimates across ages. Methods could potentially be developed to attempt to separate out consideration both of spillover effects of poor child health on caregivers and the future impacts of poor health and developmental delay in infants. In principle, explicitly asking respondents *not* to consider such things is similar to the instructions we currently give asking respondents not to take into account any possibility of treatment or alleviation of problems when evaluating states. Whether such instructions are effective across all respondents is unclear.

## Alternatives for preference-weighting EQ-TIPS

Given the issues we have identified, methods for eliciting values for EQ-TIPS could require quite different strategies than those currently used for valuing other EuroQol instruments. Alternatives to the methods currently used to value Y-3L could include the use of non-linear DCE with duration which addresses time preference [52]. These methods have been used to explore values for Y-5L [53]. Other novel valuation methods are also possible e.g. Kaizen tasks [54]. Hausman (2024) has recommended use of group-based deliberation to establish child HRQoL values [55]. Such an approach could have particular appeal in the case of valuing EQ-TIPS, given the additional challenges we have identified. Group based deliberation could include parents/caregivers, experts in child health and development and other adults, with a focus on understanding the reasoning that sits behind values and ensuring that values are meaningful.

However, if the valuation methods that are used are different, the characteristics and properties of the resulting values may also be different. This raises an important further consideration for the interpretation of values produced for EQ-TIPS, in comparison to those for older children (such as the values for Y-3L) and for adult health states. Arguably, a desirable property of values for age-specific instruments is broad comparability, in order to avoid artefactual changes in estimates of HRQoL and QALYs in cost utility analyses that entail transitions between age-specific instruments across the life span, and other applications that involve comparisons of preference-weighted HRQoL between age groups [5].

Given the complexities in eliciting stated preferences for EQ-TIPS, there may be an argument for exploring other options for establishing preference-weights for EQ-TIPS. For example, latent scale DCE can readily be used to establish the relative importance of the EQ-TIPS dimensions. These preferences can then be anchored to the 0-1 scale by using information on the position of dead = 0 obtained from Y-3L values for older children. Such an approach avoids the need to ask respondents to consider ‘life or death’ scenarios specific to 0-3-year-olds, while allowing preferences specific to EQ-TIPS to determine the relative importance of its dimensions.

An alternative to directly eliciting stated preferences for EQ-TIPS health states is to establish a response mapping (‘crosswalk’) between EQ-TIPS and the-Y-3L (or Y-5L) descriptive systems, allowing value sets for the Y instruments to be used to preference weight EQ-TIPS data. This would avoid altogether the need to obtain stated preferences data specific to EQ-TIPS. A key advantage of using a crosswak is that it would ensure the consistency of values applied to health states of infants and toddlers with those of health values for older children, avoiding discontinuities in values and QALY estimates across age-specific instruments [5] (in much the same way HuPS is linked to HUI3 utilities) [13]. Large datasets that include EQ-TIPS and Y-3L (and/or Y-5L) responses collected in parallel from proxy reporters are available (for example, from the Australian P-MIC study) [56] to facilitate exploration of the feasibility of mapping. If a crosswalk can be established, its use rests on the assumption that Y-3L values represent relevant preferences for EQ-TIPS health states, despite having been elicited in the context of health states for a 10 year old , and despite not reflecting the experience of parents and caregivers who are most familiar with health states in infants and toddlers. Mapping would therefore need to be approached with care, and such caveats made clear to those wishing to use mapped values to estimate QALYs.

## Conclusions

Valuing health states in children is challenging – and valuing health states in infants and toddlers even more so. Establishing the relative importance of EQ-TIPS dimensions is readily achieved; the principal challenge lies in anchoring these values at 0 and 1.

We conclude that eliciting stated preferences for EQ-TIPS would require considerable adaptation to the existing valuation methods used to value the EQ-5D-Y-3L. Parent-based valuation may be a viable approach, although methodological complexities remain. The nature and extent of the adaptations to valuation methods required to elicit preferences for health states in 0–3 year olds could alter the characteristics of the values such that comparability with Y-3L values is compromised. This may have implications for the use of values e.g. in the estimation of QALY gains that span infancy, childhood and adulthood. Alternative means of preference weighting EQ-TIPS—including mapping to the EQ-5D-Y (3L or 5L)—may offer a pragmatic way forward.

Finally, there is also a need to better understand users’ and decision makers’ requirements in measuring health in infants and toddlers, how EQ-TIPS data will be used, and whether age-specific value sets are required. Stakeholder engagement on these issues is therefore a essential precursor to decisions about how best to preference-weight EQ-TIPS.

## Appendix 1

EQ-TIPS-3L (EuroQol Toddler and Infant Populations Questionnaire for children 0–3 years), English Experimental version, version 3.

© 2021 EuroQol Research Foundation. EQ-TIPS™ is a trademark of the EuroQol Research Foundation. Generic English v3.0 Experimental. Reproduced by permission of EuroQol Resarch Foundation. Reproduction is not allowed. For the reproduction, use or translation of the EQ-TIPS (any version), please contact EuroQol’s R&D Manager: rd@euroqol.org. EQ-TIPS has an Experimental Version status: It is only shared in the context of a collaboration for the development of the Experimental Version. It is not a final product and is subject to change.

© 2021 EuroQol Research Foundation. EQ-TIPS™ is a trademark of the EuroQol Research Foundation. Generic English v3.0 Experimental. Reproduced by permission of EuroQol Resarch Foundation. Reproduction is not allowed. For the reproduction, use or translation of the EQ-TIPS (any version), please contact EuroQol’s R&D Manager: rd@euroqol.org. EQ-TIPS has an Experimental Version status: It is only shared in the context of a collaboration for the development of the Experimental Version. It is not a final product and is subject to change.

## Footnotes

1 Further, as EQ-TIPS is never self-completed by children in the 0–3 years range, its use as a measure of HRQoL relies on proxy completion. The use of parents to both report *and* value health states may have some appeal to consistency, although the parallel in adult populations (i.e. asking patients to both report and value their health) is not considered compelling by many HTA bodies.

2 In the language of economics, Proxy 2 requires that respondents (for example, parents) act as ‘perfect agents’ for their child, and have complete knowledge of their child’s preferences.

3 Other than at the most basic level e.g. less pain is preferred to more pain. Such preferences provide no more information than is already possible by analysis of the profile data (e.g. lower levels of pain are preferred to higher levels of pain).

4 It may be worth noting that, in its use to *report* health in 0–3 year olds, EQ-TIPS only uses a ‘Proxy 1’ approach (responding to the questionnaire by providing their own impression of the respondent’s health) for similar reasons.

5 However, it might be noted that in valuing adult EQ-5D-5L we generally don’t seek stated preferences from those < 18 years, so it is not correct to say the EQ-5D-5L values reflect the average preferences of ‘society’ – the values represents the views of the *adult* general public, which is a sub-set of society.
